# Identification of *Arabidopsis Phospholipase A* Mutants With Increased Susceptibility to *Plasmodiophora brassicae*

**DOI:** 10.3389/fpls.2022.799142

**Published:** 2022-02-18

**Authors:** Qinqin Zhou, Kethmi N. Jayawardhane, Stephen E. Strelkov, Sheau-Fang Hwang, Guanqun Chen

**Affiliations:** Department of Agricultural, Food and Nutritional Science, University of Alberta, Edmonton, AB, Canada

**Keywords:** clubroot, *Arabidopsis*, canola, *phospholipase A*, *PLA* genes, resistance

## Abstract

Clubroot, caused by the obligate parasite *Plasmodiophora brassicae*, is one of the most devastating diseases of canola (*Brassica napus*) in Canada. The identification of novel genes that contribute to clubroot resistance is important for the sustainable management of clubroot, as these genes may be used in the development of resistant canola cultivars. Phospholipase As (PLAs) play important roles in plant defense signaling and stress tolerance, and thus are attractive targets for crop breeding. However, since canola is an allopolyploid and has multiple copies of each *PLA* gene, it is time-consuming to test the functions of *PLAs* directly in this crop. In contrast, the model plant *Arabidopsis thaliana* has a simpler genetic background and only one copy of each *PLA*. Therefore, it would be reasonable and faster to validate the potential utility of *PLA* genes in *Arabidopsis* first. In this study, we identified seven homozygous *atpla* knockout/knockdown mutants of *Arabidopsis*, and tested their performance following inoculation with *P. brassicae*. Four mutants (*pla_1_-ii*α, *pla_1_-i*γ*3*, *pla_1_-iii*, *ppla-iii*β, *ppla-iii*δ) developed more severe clubroot than the wild-type, suggesting increased susceptibility to *P. brassicae*. The homologs of these *Arabidopsis PLAs* (*AtPLAs*) in *B. napus* (*BnPLAs*) were identified through Blast searches and phylogenic analysis. Expression of the *BnPLAs* was subsequently examined in transcriptomic datasets generated from canola infected by *P. brassicae*, and promising candidates for further characterization identified.

## Introduction

Clubroot, caused by the obligate parasite *Plasmodiophora brassicae*, is one of the most devastating diseases of canola (oilseed rape; *Brassica napus*) in Canada and other regions. The infected host plants develop characteristic root galls, which interfere with water and nutrient uptake, leading to aboveground symptoms including yellowing and wilting of the leaves, stunting, premature ripening, and losses in seed yield and quality (Pageau et al., 2006; Hwang et al., 2012). The deployment of clubroot resistant (CR) cultivars has been the main strategy to manage clubroot in western Canada (Hwang et al., 2014). Unfortunately, the effectiveness of resistance can be quickly lost as a result of the selection pressure imposed on pathogen populations by short rotations with CR cultivars carrying similar sources of resistance. In Canada, “new” pathotypes of *P. brassicae* able to overcome the resistance in most CR canola cultivars were identified in 2013, only 4 years after the introduction of the resistance trait (Strelkov et al., 2016). Since then, resistance-breaking pathotypes, many of which exhibit novel virulence patterns, have documented in many fields (Strelkov et al., 2018; Hollman et al., 2021). Given these rapid shifts in the virulence of pathogen populations, it is necessary to identify additional sources of resistance, including novel gene targets in canola (Zhou et al., 2020a), to aid in sustainable clubroot management. These could be used in rotations with existing CR canola cultivars, helping to reduce selection pressure on pathogen populations and contributing to resistance stewardship (Hwang et al., 2019).

Phospholipase As (PLAs), which catalyze the hydrolysis of membrane phospholipids into free fatty acids and lysophospholipids, are attractive targets for crop breeding due to their important roles in plant defense signaling and stress tolerance (Yang et al., 2007; Canonne et al., 2011; Chen et al., 2013). PLAs are involved in the plant response to biotic stress by inducing jasmonic acid (JA), oxylipin and phytoalexin biosynthesis (Canonne et al., 2011; Ruelland et al., 2015). In addition, PLA activity was suggested to be important in elicitor-induced oxidative burst (e.g., *Verticillium dahliae* extract) (Chandra et al., 1996). Recent transcriptomic studies indicate the involvement of phospholipases in the host response to *P. brassicae*. For example, the expression of many genes encoding phospholipases was increased in the roots of *P. brassicae*-infected *Arabidopsis* at 24 days after inoculation (dai) (Irani et al., 2018). Expression profiling of *B. napus* transcriptome datasets enabled the identification of differentially expressed genes, including *PLA*s, following *P. brassicae* inoculation (Galindo-González et al., 2020; Zhou et al., 2020b).

Since PLAs appear to contribute to the response of host plants to pathogens, functional validation of their role against *P. brassicae* will provide valuable information regarding their potential utility in the development of CR canola cultivars. However, since the model plant *Arabidopsis thaliana* (*Arabidopsis* thereafter) possesses over 20 PLAs and canola has several homologs of each of these genes (Iqbal et al., 2020), it is time-consuming to test the functions of *BnPLAs* directly in canola. In contrast, *Arabidopsis* has a simpler genetic background (Cheng et al., 2014) and only one copy of each *PLA* gene (Chen et al., 2013), and as a crucifer, also serves as a host of *P. brassicae*. Therefore, it would be more feasible and faster to validate the roles of *PLA* genes in clubroot resistance in *Arabidopsis*, to select promising candidates for further characterization in canola.

In this study, we identified and selected seven T-DNA insertion *pla* knockout/knockdown mutants of *Arabidopsis* from the *Arabidopsis* Biological Resource Center, and compared their performance with the wild-type plants following inoculation with *P. brassicae*. We believe that, this study would be first step in characterizing the role of *PLAs* in clubroot resistance, and will serve as the foundation of additional work in this area. The results showed that four mutants (*pla_1_-ii*α, *pla_1_-i*γ*3*, *pla_1_-iii*, *ppla-iii*δ) developed more severe clubroot than the wild-type, suggesting increased susceptibility to *P. brassicae*. After identifying the homologs of the seven *AtPLAs* in canola (*BnPLAs*), and examining their expression profiles in published transcriptomic datasets generated from *B. napus* inoculated with *P. brassicae*, several *BnPLAs* were identified as promising candidates for further characterization in canola.

## Materials and Methods

### Plant Materials and Growth Conditions

The *Arabidopsis* mutants *pla_1_-i*γ*1* (CS855673), *pla_1_-ii*α (SALK_086894C), *pla_1_-i*γ*3* (SALK_012432C), *pla_1_-iii* (SALK_033291), *spla_2_-*α (SALK_099415C), *ppla-iii*β (SALK_057212C) and *ppla-iii*δ (SALK_029470), derived from the wild-type ecotype Columbia, were obtained from the *Arabidopsis* Biological Resource Center (abrc.osu.edu; Table 1). The seeds were multiplied by growing into the next generation, and the homozygosity of T-DNA insertion of all the *pla* mutants was confirmed by PCR using two gene-specific primers (LP and RP) and a T-DNA border primer (Table 2). The seed was multiplied in a growth chamber set at 22/18°C with a photoperiod of 16 h day/8 h night. For inoculation with *P. brassicae*, all of the *pla* mutants and Columbia were grown in Sunshine LA4 potting mix (SunGro Horticulture, Vancouver, BC, Canada) in a greenhouse under 16 h light (natural light supplemented by artificial lighting) at 22°C. Three to five cold stratified (at 4°C for 3 days) seeds were placed in each pot (6 cm × 6 cm × 6 cm) and were thinned to one plant per pot after 1 week.

**TABLE 1:** *Arabidopsis thaliana* (At) *PLAs* included in this study and their homologs in *Brassica napus* (Bn).

Germplasm	Gene	At gene ID	Bn gene ID	Bn peptide ID	Identity (%)	Coverage (%)	*E*-value
CS855673	*PLA1-I*γ*1*	AT1G06800	BnaC05g04710D	CDY10228	86.65	99.22	0
			BnaA10g27930D	CDY54880	85.39	83.88	0
SALK_086894C	*PLA1-II*α	At1G06250	BnaC05g04300D	CDY10187	83.08	94.80	0
			BnaA10g28020D	CDY57813	82.84	94.80	0
			BnaC08g01730D	CDX86530	81.59	94.80	0
			BnaA08g28540D	CDY15570	81.48	95.51	0
SALK_012432C	*PLA1-I*γ*3*	AT1G51440	BnaA06g02350D	CDY22842	83.85	98.48	0
			BnaC06g04560D	CDY49813	82.50	98.48	0
SALK_033291	*PLA1-III*	AT1G30370	BnaA07g07350D	CDX77597	93.22	80.15	0
			BnaC05g23260D	CDY09593	87.15	99.81	0
			BnaA09g26150D	CDY02287	86.96	99.81	0
SALK_099415C	*sPLA2-*α	AT2G06925	BnaA05g14430D	CDY25852	85.33	99.32	1E–84
			BnaCnng35860D	CDY60208	85.33	99.32	1E–84
SALK_057212C	*pPLA-III*β	AT3G54950	BnaC08g25800D	CDX73423	86.17	99.80	0
			BnaA07g16580D	CDX67618	84.66	99.80	0
			BnaA09g55020D	CDY56960	84.20	99.80	0
			BnaC06g15520D	CDY07512	82.93	99.80	0
SALK_029470	*pPLA-III*δ	AT3G63200	BnaA04g00050D	CDX89181	87.53	99.74	0
			BnaC04g20810D	CDX93922	87.02	99.74	0
			BnaC08g32750D	CDX76689	85.46	99.22	0
			BnaA09g55720D	CDY56455	81.63	99.22	0

**TABLE 2:** Primers used for T-DNA homozygous mutant lines identification.

Gene ID	Mutant line	Right genomic primer	Left genomic primer	T-DNA border primer
At1g06800	WiscDsLox434H9	CGATTTCGATCCATTTTCAAG	TTATTCTCCATTCGGCATTTG	
At1g06250	SALK_086894	TCTACAGCCATATGAATGGGC	ATTCGGTTCCCAGATTCTTTG	
At1g51440	SALK_012432	ATGACTATGTCACGTCTCCCG	TTATTACCCATCCACGATCCC	
At1g30370	SALK_033291	TGTGGAAGAGGTGTTTCTTGG	AAATGCAAGTTCAATTGTTCG	
At2g06925	SALK_099415	ACTGTCCACTTCATGATTGCC	TTTGTTACCTTTGAACGTCGG	
At3g54950	SALK_057212	ATAAACCGGTTAAATCCACCG	GTTACTACTGCGGCAGTGACC	
At3g63200	SALK_029470	CGTCGATGCTAAGGATACGAG	TACTCACACGTGCGAGAACAG	
For SALK lines (LBb1.3)				GCGTGGACCGCTTGCTGCAACT
For WISCDSLOX (P745)				AACGTCCGCAATGTGTTATTAAGTTGTC

### Pathogen Material and Inoculation

Inoculations were conducted with a single-spore isolate of *P. brassicae* pathotype 3H, as classified on the Canadian Clubroot Differential set (Strelkov et al., 2018), which had been stored at −20°C in infected root galls of the *B. napus* cultivar “Brutor.” The resting spore inoculum suspension was prepared following Strelkov et al. (2006). Briefly, 5 g of the frozen galls were homogenized in 100 mL water and filtered through eight layers of cheesecloth to remove plant debris. The resting spore concentration was measured with a hemocytometer and adjusted to 1.0 × 10^5^ resting spores/mL with sterile distilled water. Two-week old seedlings were inoculated by modifying the method of Ludwig-Müller et al. (2017). Briefly, 2 mL of the inoculum suspension was added to the soil near the base of each plant with pipette. The non-inoculated control plants were treated with same amount of water using the same method.

The severity of clubroot was evaluated at 3 to 4 weeks after inoculation when the aboveground parts of inoculated plants were yellowing and purpling. Treatments were replicated six times with 24 plants per replicate. Clubroot severity was evaluated on a 0–3 scale (Figure 2A), where: 0 = no galls, 1 = galls mainly on the lateral roots, 2 = obvious galls on both the primary and lateral roots with a moderately reduced root system, and 3 = large galls on the primary roots with a significantly reduced root system. The individual severity ratings were used to calculate a disease index (DI) for each replicate using the formula described by Strelkov et al. (2006): DI (%) = [(n_1_ × 1 + n_2_ × 2 + n_3_ × 3)/(N × 3)] × 100, where n_1_, n_2_, and n_3_ are the number of plants in each severity class and N are the total number of plants tested. An one-way Anova followed by the Dunnett’s test in R (r-project.org) was used to compare the mean DIs of each *Arabidopsis* mutant to the wild-type, with differences regarded as significant at *p* < 0.05.

### Identification of AtPLA Homologs in *Brassica napus*

Homologs of the seven *AtPLAs* from *Arabidopsis* were identified in *B. napus* through BLAST and phylogenic analysis. Specifically, peptide sequences of the seven *AtPLA* obtained from the *Arabidopsis* (TAIR 11) database^1^ were blasted against the *B. napus* genome (Chalhoub et al., 2014) using BLASTP (*E*-value ≤1e–10, coverage >60%, identity >60%, and the top 20 hits).^2^ Homologs of the *AtPLAs* also were identified in *B. rapa* and *B. oleracea* using the same parameters, to improve the accuracy of the subsequent phylogenic analysis. The sequences of selected peptides of the three *Brassica* species were aligned with the seven AtPLA peptide sequences using Muscle in MEGA7 (Kumar et al., 2016). A rooted phylogenic tree of the selected PLA peptides in the four species, using *WRKY2* (AT5G56270) in *Arabidopsis* as the root, was generated with fasttress (Price et al., 2009) and visualized in MEGA7 to identify the *AtPLA* homologs in *B. napus*. In addition we have identified pylogenic relationship between all the available *Arabidopsis* PLAs and *Brassica* species. The expression profiles of the *BnPLAs* were examined in two published transcriptomic datasets generated from *B. napus* infected by *P. brassicae* (Galindo-González et al., 2020; Zhou et al., 2020b; Supplementary Figure 2).

## Results

**FIGURE 1:**
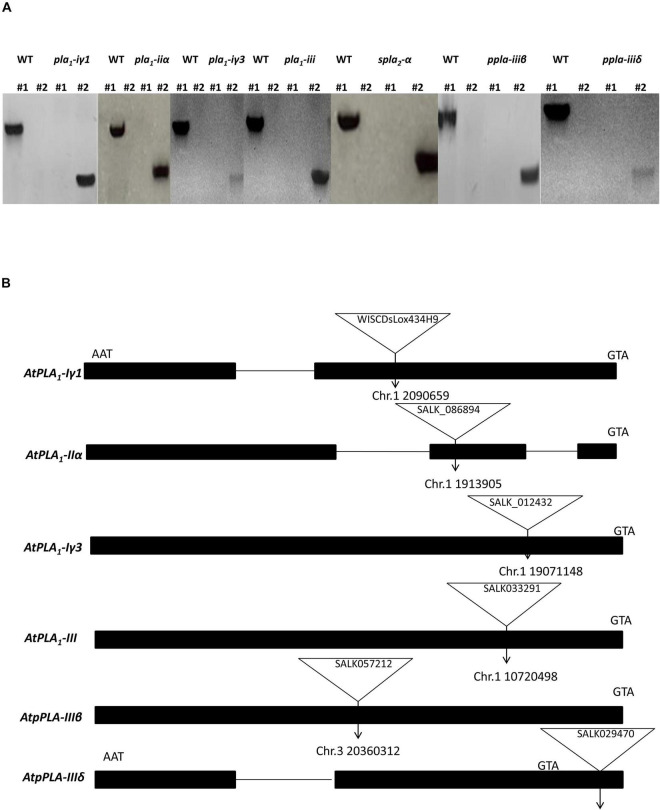
Identification and characterization of *Arabidopsis* T-DNA insertion lines. **(A)** Identification of T-DNA insertion lines by PCR. LP and RP primers are located at the left and right sides of T-DNA insertion, respectively. LBb1.3 and P745 are used as flanking primers for SALK and WiSC lines, respectively. In each gel picture #1 is the PCR sample with primers LP + RP and #2 is the PCR sample with primers LB1.3/P745 + RP. **(B)** Schematic diagrams of the positions of T-DNA insertions in PLA single mutant alleles. Start and stop codons are indicated.

We identified homozygous T-DNA insertion knockout/knockdown mutants representing each PLA subtype, *pla_1_-i*γ*1* (WiscDsLox434H9), *pla_1_-ii*α (SALK_086894C), *pla_1_-i*γ*3* (SALK_012432C), *spla_2_-*α (SALK_099415C), *ppla-iii*β (SALK_057212C), *pla_1_-iii* (SALK_033291), and *ppla-iii*δ (SALK_029470), using two gene-specific primers and the T-DNA left border primers (Alonso et al., 2003; Figure 1A). Insertion sites of *pla_1_-i*γ, *pla_1_-ii*α, *pla_1_-i*γ*3*, *ppla-iii*β, and *pla1-iii* were located in the exon regions of each *PLA* genes, while *ppla-iii*δ contained its mutant sites within the promoter region (Figure 1B). Furthermore, while the *spla_2_-*α mutant lines were reported to have multiple insertion sites within the promoter and exon regions of the gene, the exact map location was not indicated on the TAIR web site (Seo et al., 2008; TAIR-ABRC). Furthermore, this T-DNA mutant line (SALK_099415C) previously been confirmed as a complete gene knockout line as no detectable mRNA was observed (Seo et al., 2008).

**FIGURE 2:**
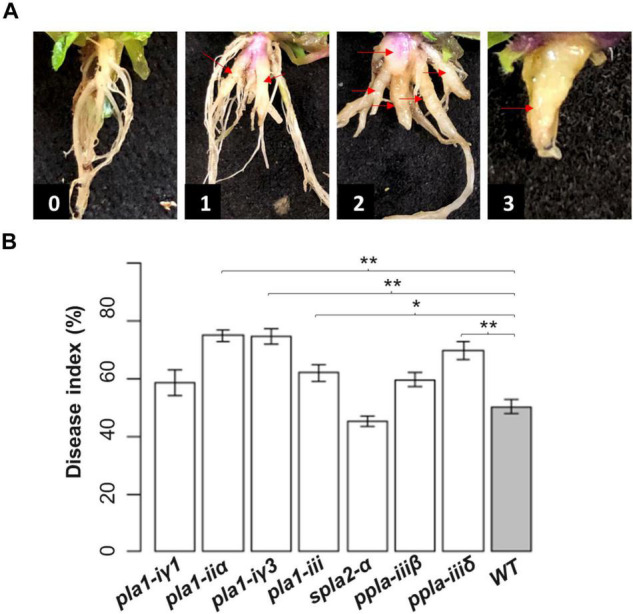
Evaluation of reaction of seven *pla* mutants and wild-type (Columbia) *Arabidopsis* to inoculation with *Plasmodiophora brassicae* pathotype 3H. **(A)** The rating scale used to evaluate clubroot disease severity, where: 0 = no galls, 1 = galls mainly on the lateral roots, 2 = obvious galls on both the primary and lateral roots with a moderately reduced root system, and 3 = large galls on the primary roots with a significantly reduced root system. The individual severity ratings were used to calculate a disease index. **(B)** The disease index on the seven *pla* mutants and the wild-type Columbia 26 days after inoculation with *P. brassicae*. Values are means ± SE of six independent replicates with 24 plants per replicate. The asterisks indicate significant differences at *p* < 0.05 (*) and *p* < 0.01 (**) compared with the wild-type, based on the Dunnett’s test in R.

To validate the roles of the selected *PLAs* in the host response to clubroot, we first evaluated the disease severity of the seven homozygous *Arabidopsis pla* mutants and the wild-type *Arabidopsis*, following inoculation with *P. brassicae* pathotype 3H. Four of the mutants (*pla_1_-ii*α, *pla_1_-i*γ*3*, *pla_1_-iii*, and *ppla-iii*δ) had significantly greater DIs than the wild-type (Figure 2B), indicating increased susceptibility to *P. brassicae* pathotype 3H. Among these, the *pla_1_-ii*α and *pla_1_-i*γ*3* appeared the most susceptible, with DIs of 75.0 and 74.7%, respectively, followed by *ppla-iii*δ (69.8%) and *pla_1_-iii* (62.1%). The reactions for the remaining mutants were similar to the wild-type, which developed an DI of 50.4%.

**FIGURE 3:**
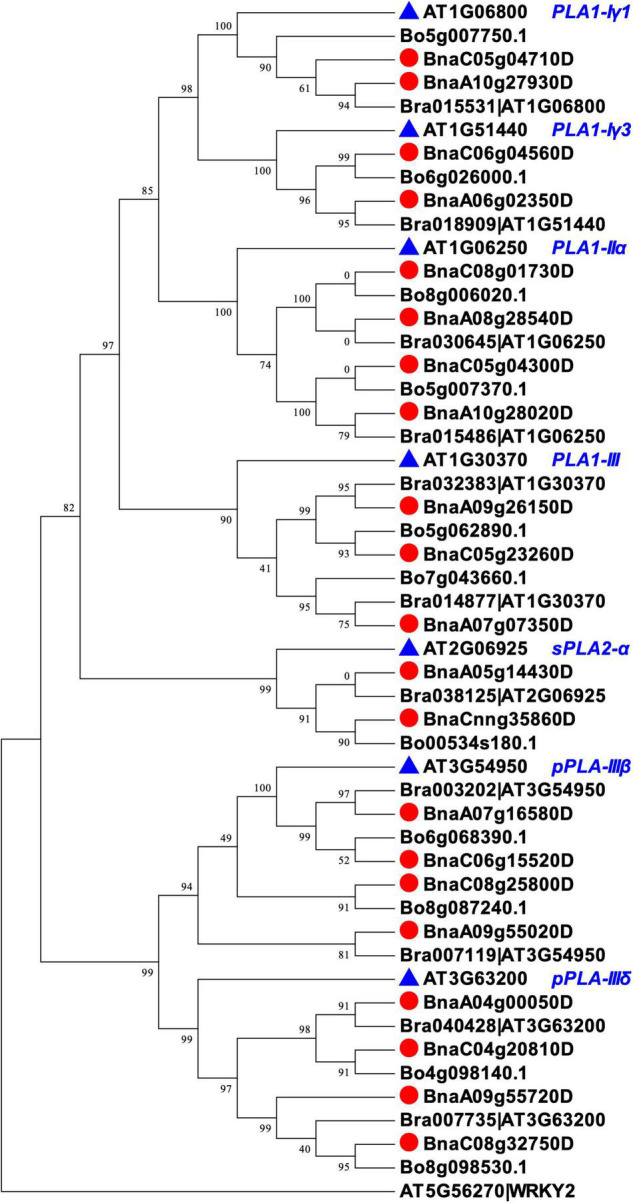
Phylogenic relationships of PLAs in *Arabidopsis* and *Brassica* species (*B. rapa*, *B. oleracea*, and *B. napus*). The phylogenic tree was constructed with fasttree using PLA peptide sequences aligned by MUSCEL in Mega7. *Arabidopsis* WKRY2 (AT5G56270) was used as the root. Each PLA encoding gene in *B. rapa* was followed with an *Arabidopsis* gene ID in the genome annotation, which was kept in the phylogenic tree to confirm the clustering of the PLAs.

We identified homologs of the seven *PLAs* in *B. napus* by BLAST and phylogenic analysis. We first aligned the peptide sequences of seven AtPLA to the three *Brassica* species (*B. rapa*, *B. oleracea*, and *B. napus*) using BLASTP (see text footnote 2) to select potential *PLA* homologs with high identity (%) and coverage (%) and low *E*-values. After that, we performed phylogenetic analysis using Mega7 (Kumar et al., 2016) and fasttree (Price et al., 2009), and filtered *Brassica PLAs* that were not properly clustered with the targeted *AtPLAs*. In total, 21 homologs of *BnPLAs* were identified in *B. napus*, which were evolved from either *B. rapa* or *B. oleracea* (Figure 3), indicating duplication and loss of *PLAs* during the divergence of *Arabidopsis* and *Brassica*, but a conserved evolution of *PLAs* in *Brassica*. All the *BnPLAs* had >80% identity and coverage, and *E*-values near 0, to each of corresponding *AtPLA* (Table 1).

## Discussion

Plant phospholipids are major structural components of biological membranes (Adigun et al., 2021). Accumulating evidence indicates that phospholipid-based signal transduction and phospholipid-derived products mediated by PLAs are important for plant growth, development, and responses to abiotic and biotic stress (Ryu, 2004; Grienenberger et al., 2010; Canonne et al., 2011; Chen et al., 2013; Adigun et al., 2021). Upon perception of invading pathogens, PLAs can be activated to hydrolyze phospholipids and galactolipids at their *sn*-1 or *sn*-2 positions to generate free fatty acids and lysophospholipids. These function as precursors of second messengers to mediate downstream defense reactions, including the production of oxylipins and JA, and stimulation or inhibition of key signaling enzymes (e.g., MAKP, protein kinase and H^+^-ATPase) (Ryu, 2004; Canonne et al., 2011). PLAs are also involved in plant growth and development by auxin-related cell elongation in plants (Ryu, 2004), which might be associated with root galling in *P. brassicae*-infected plants. In addition, a recent expression analysis of drought-related lipid genes in soybean (*Glycine max*) suggested the involvement of *PLAs* (*PLA_1_-II* and *-III*, *sPLA*, *pPLA-I*, *-II*, and *-III*) in response to water deficit (Ferreira, 2017). Since infection by *P. brassicae* interferes with root water uptake and leads to drought stress symptoms in the aboveground canopy (Ludwig-Müller, 2009), PLAs might be involved in a clubroot-related drought response in plants. While little is known regarding the role of PLAs in the host response to clubroot, recently published transcriptomic profiles from *P. brassicae*-infected *B. napus* allowed us to identify *PLAs* showing differential expression following inoculation (Fu et al., 2019; Galindo-González et al., 2020; Zhou et al., 2020b). Therefore, study of the role(s) of PLAs in the host response to clubroot could provide insights into the utility of PLAs in clubroot resistance breeding.

In plants, PLAs are classified into three main subtypes based on their catalysis site: phospholipase A1 (PLA_1_), secretory phospholipase A2 (sPLA_2_), and patatin-like PLA (pPLA). *Arabidopsis* has about 30 PLAs, including 12 PLA_1_s, four sPLA_2_s, and 13 pPLAs (Eastmond, 2006; Chen et al., 2013; Li and Wang, 2014). Furthermore, in canola, each PLA has several homologs (Supplementary Figure 1) and it is difficult to study their roles in clubroot resistance directly given the complexity of the canola genome. To improve understanding of the roles of PLAs in clubroot resistance and to help to identify candidate *PLA* genes in canola contributing to resistance, we selected seven *pla* knockout/knockdown *Arabidopsis* mutants and compared their susceptibilities with the wild-type plants, following inoculation with *P. brassicae* pathotype 3H. This is a common pathotype found across much of the canola producing regions of western Canada, and is highly virulent on canola that does not carry any clubroot resistance (Strelkov et al., 2018; Hollman et al., 2021).

Phospholipase A1s specifically hydrolyze the *sn-1* position in phospholipids. They further divided into groups I, II, and III PLA_1_s and phosphatidic acid-specific PLA_1_ (PA-PLA_1_) (Kim and Ryu, 2014). Class I, II, and III PLA_1_s are predicted to localize to the chloroplast, cytosol and mitochondria, respectively, based on the presence of N-terminal stretches (Ryu, 2004; Seo et al., 2009; Chen et al., 2011). While there is no clear evidence of PLA_1_s in plant immunity, expression of several genes (e.g., *PLA_1_-I*γ*1*, *PLA_1_-I*γ*2*, and *PLA_1_-III*) encoding PLA_1_ proteins was induced in *Arabidopsis* response to pathogen attack (e.g., *B. cinerea* and *P. syringae*) (Grienenberger et al., 2010). In addition, increased expression of several PLA_1_s (*VviPLA_1_-I*β*1*, *VviPLA_1_-I*γ*1*, and *VviPLA_1_-II*δ) in grapevine (*Vitis vinifera* L.) was found within 24 h following inoculation with the biotrophic oomycete *Plasmopara viticola* (Laureano, 2018). Three of the four *pla*_1_ mutants (*pla_1_-ii*α, *pla_1_-i*γ*3*, and *pla_1_-iii*) in the current were more susceptible to *P. brassicae* than the wild-type *Arabidopsis*, indicating the importance of *PLA*_1_ in host defense to this pathogen.

Class I *PLA*_1_s are involved in JA production (Wasternack and Song, 2017), including the two genes *PLA_1_-I*γ*1* and *PLA_1_-I*γ*3* in this study (Ellinger et al., 2010). However, the genes may mediate different pathways for JA production, with *PLA_1_-I*γ*1* contributing to wound-induced JA production and *PLA_1_-I*γ*3* contributing to JA production under non-wounded conditions (Ellinger et al., 2010). Transcription of *PLA_1_-I*γ*1* in *Arabidopsis* was induced upon *B. cinerea* and *P. syringae* inoculation (Grienenberger et al., 2010), and expression of *PLA_1_-I*γ*1* in grapevine was upregulated within 24 h of inoculation with *P. viticola* (Laureano, 2018). A homolog of *AtPLA_1_-I*γ*1* in *B. napus* was downregulated (BnaC05g04710D) during the late infection stage in a compatible (susceptible) interaction with *P. brassicae* pathotype 3A (Zhou et al., 2020b), while the same gene was upregulated during early infection of a resistant *B. napus* genotype by pathotype 5X (Galindo-González et al., 2020; Supplementary Figure 2). Galindo-González et al. (2020) also identified another *BnPLA_1_-I*γ*1* (BnaA10g27920D), which was upregulated in both susceptible and resistant *B. napus*, but with greater upregulation in the resistant reaction. In this study, *pla1-i*γ*1* and *pla1-i*γ*3* mutants showed greater susceptibility than the wild-type, but only *pla1-i*γ*3* showed a significant difference, suggesting that *PLA_1_-I*γ*3* but not *PLA_1_-I*γ*1* plays an important role in the response of *Arabidopsis* to *P. brassicae*. Thus, homologs of *PLA_1_-I*γ*3* in canola may contribute to enhanced clubroot resistance. Given the fact that *BnPLA_1_-I*γ*1* (BnaC05g04710D) was downregulated in a clubroot susceptible reaction and upregulated in a resistant reaction, this gene might also be associated with canola defense to clubroot.

There are four class II PLA_1_s in *Arabidopsis* (PLA_1_-IIα, -IIβ, -IIγ, and -IIδ) (Chen et al., 2013) but little is known regarding the role of class II PLA_1_s in response to pathogens. Nonetheless, the expression of *PLA_1_-II*δ increased in grapevine following challenge by *P. viticola* (Laureano, 2018), suggesting the involvement of class II PLA_1_s in the defense reaction. Our results showed that the *pla_1_-ii*α *Arabidopsis* mutant was more susceptible to clubroot than the wild-type, suggesting a role for *PLA_1_-II*α in the host response to *P. brassicae*. In addition, a homolog of this gene in *B. napus* (BnaA10g28020D) was downregulated during the late stage of susceptible reaction to clubroot (Zhou et al., 2020b), which is consistent with the present results. Since a *PLA_1_-II* (*GmPLA1-II*γ*a*) in soybean was upregulated under drought conditions (Ferreira, 2017), *PLA_1_-II*α-mediated clubroot defense might include mitigating water stress. Collectively, these results suggest the importance of *PLA1-II*α in clubroot resistance, and the gene BnaA10g28020D might be a good candidate for further characterization of its role.

Only one class III PLA_1_ is found in *Arabidopsis* (PLA_1_-III) (Chen et al., 2013). PLA_1_-III is important for plant development and stress tolerance. The expression of *PLA_1_-III* was modulated in *Arabidopsis* upon *B. cinerea* (Grienenberger et al., 2010) challenge, respectively. Expression of an *AtPLA_1_-III* homolog in CR Chinese cabbage (*B. rapa*) was induced (BraA07g010560.3C) during the early infection stage (4 dai) following inoculation with a virulent pathotype of *P. brassicae* (Fu et al., 2019). A homolog of *AtPLA_1_-III* was downregulated in *B. napus* (BnaC05g23260D) during the late stage of the clubroot-susceptible response (Galindo-González et al., 2020; Zhou et al., 2020b; Supplementary Figure 2), while two other *BnPLA_1_-III* homologs (BnaA07g07350D and BnaA09g26150D) were downregulated in both resistant and susceptible reactions, with greater downregulation in the latter (Galindo-González et al., 2020). These findings are consistent with our results, since the *pla_1_-iii* mutant was more susceptible to clubroot than the wild-type, indicating the importance of PLA_1_-III in plant defense to clubroot. Similarly, *PLA_1_-III* in soybean was regulated under drought conditions, with a downregulation in moderate and severe drought but an upregulation in extreme drought (Ferreira, 2017), further suggestion a role in mitigating drought stress.

sPLA_2_s, which specifically hydrolyze the *sn-2* position of phospholipids, are the only PLA_2_s identified in plants (Kim and Ryu, 2014). Only four AtsPLA isoforms are found in *Arabidopsis* (sPLA_2_-α, -β, -γ, and -δ), with sPLA_2_-α suggested to play a negative role in defense to pathogen attack by subcellular localizing to the cell nucleus and physically binding to MYB30 to repress defense in *Arabidopsis* (Froidurea et al., 2010; Canonne et al., 2011; Kim and Ryu, 2014). sPLA_2_ have been suggested to be involved in the auxin signaling pathway, which promotes cell elongation (Ryu, 2004). For instance, overexpression of *AtsPLA_2_-*β in *Arabidopsis* resulted in elongation of the leaf petioles and inflorescence stems, while silencing of the gene resulted in the opposite phenotype (Lee et al., 2003). Expression of *BnsPLA_2_-*α was increased in susceptible hosts and decreased in resistant hosts, following inoculation with *P. brassicae* (Galindo-González et al., 2020; Zhou et al., 2020b; Supplementary Figure 2), suggesting their negative roles in clubroot resistance. Considering that auxin is a crucial hormone for gall development in roots of *P. brassicae*-infected plants (Jahn et al., 2013), *sPLA_2_-*α might be involved in auxin-related pathways to promote gall development. Nonetheless, in this study, clubroot severity on the *atspla_2_-*α mutant was not significantly different from that of the wild-type.

Patatin-like PLAs, which hydrolyze phospholipids and galactolipids at both the *sn-1* and *sn-2* positions (Chen et al., 2011), are involved in defense signaling in plants upon infection by many pathogens. A member of pPLAs in pepper (*Capsicum annuum*), CaPLP1, plays a positive role in plant innate immunity (Kim et al., 2014). Silencing of *CaPLP1* increased plant susceptibility to the bacterium *Xanthomonas campestris* pv. *vesicatoria* and was associated with compromised defense responses, including reactive oxygen species production, hypersensitive cell death and expression of a SA-dependent gene *CaPR1*. Overexpression of this gene in *Arabidopsis* increased plant resistance to *P. syringae* and *Hyaloperonospora arabidopsidis*, which was associated with an enhanced oxidative burst, expression of SA- and JA-dependent genes, and cell death (Kim et al., 2014). Another pPLA, *pPLA-II*α*/PLP2*, which contributes JA- and oxylipin-mediated cell death, positively regulated *Arabidopsis* resistance to the obligate parasite *cucumber mosaic virus* and the fungus *V. dahliae* by inducing oxylipins and JA biosynthesis, respectively. This gene, however, negatively regulated resistance to *B. cinerea* and *P. syringae* in *Arabidopsis* (la Camera et al., 2005, 2009; Zhu et al., 2021). In addition, the expression of several pPLAs (*VvipPLA-I*, *VvipPLA-II*β, *VvipPLA-II*δ*2*, and *VvipPLA-III*β) was induced in grapevine after inoculation with *P. viticola* (Laureano, 2018). Transcriptomic studies of *B. napus* inoculated with *P. brassicae* pathotype 3A indicated that expression of two copies of *BnPLP2* increased earlier during the infection process, but not at later stages, in both the resistant and susceptible reactions (Zhou et al., 2020b). When *B. napus* was inoculated with *P. brassicae* pathotype 5X, expression patterns of the two genes were generally similar in both the resistant and susceptible reactions, except that the genes were also upregulated in the susceptible host during the late infection stage (Galindo-González et al., 2020; Supplementary Figure 2), suggesting a negative role of this genes in clubroot resistance. In addition, pPLAs are involved in the regulation of auxin-related responses. A study investing the role of pPLAs in the regulation of auxin responses found delayed upregulation of auxin-responsive gene expression in all nine *ppla* mutants studied (Labusch et al., 2013). The authors also demonstrated that knocking out/down *pPLA-III*δ in *Arabidopsis* affected auxin-related phenotypes, including shortened main roots and more lateral roots, and knocking out of *pPLA-III*β resulted in slightly longer roots and hypocotyls (Labusch et al., 2013).

Other pPLA enzymes, including PLAIVA/PLP1, are also positively associated with plant auxin signaling to modulate root development (Rietz et al., 2010). In addition, p*PLAs* are involved in response to water deficit in plants (Ferreira, 2017). Therefore, pPLAs could regulate the host response to clubroot *via* their involvement in defense pathways and drought- and auxin-related pathways. In this study, both *ppla-iii*β and *ppla-iii*δ appeared to develop more severe symptoms than the wild-type *Arabidopsis*, but DI was significantly more severe only for *ppla-iii*δ (Figure 2B), indicating a positive effect of *pPLA-III*δ in clubroot resistance. In constrast, *B. napus* inoculated with pathotype 3A or 5X of *P. brassicae* showed reduced expression of *BnpPLA-III*δ in both the resistant and susceptible reactions during early infection, and was downregulated in only the resistant hosts during late infection (Galindo-González et al., 2020; Zhou et al., 2020b; Supplementary Figure 2). These contrasting findings may reflect host-pathotype specific interactions, and suggest that functional validation of *AtpPLA-III*δ in response to additional *P. brassicae* pathotypes may improve understanding of its role in host defense.

## Conclusion

To the best of our knowledge, this is the first study investigating the host response of PLAs to clubroot. We selected seven *Arabidopsis atpla* knockout/knockdown mutants, and identified four mutants (*pla_1_-ii*α, *pla_1_-i*γ*3*, *pla_1_-iii*, *ppla-iii*δ) that were more susceptible to *P. brassicae* than the wild-type plants. These results indicate that *PLAs* may play positive roles in host defense to clubroot. We identified homologs of the seven PLAs in *B. napus* and its parental species (*B. rapa* and *B. oleracea*) and explored their expression patterns following *P. brassicae* inoculation using available transcriptomic datasets; this information was used to select candidate genes that can be further characterized in canola. The pathways mediated by these PLAs are unknown, but could be related to JA biosynthesis, and auxin- and drought-responses. However, additional studies will be needed with more PLA mutants in *Arabidopsis* to confirm their role in clubroot resistance and elucidate the precise mechanism(s) of each PLA gene.

## Data Availability Statement

The original contributions presented in the study are included in the article/Supplementary Material, further inquiries can be directed to the corresponding author.

## Author Contributions

SS, S-FH, and GC conceived, designed and supervised the experiment, and edited the manuscript. QZ and KJ performed the experiments and wrote the first draft of the manuscript. All authors contributed to the preparation of the final article.

## Conflict of Interest

The authors declare that the research was conducted in the absence of any commercial or financial relationships that could be construed as a potential conflict of interest.

## Publisher’s Note

All claims expressed in this article are solely those of the authors and do not necessarily represent those of their affiliated organizations, or those of the publisher, the editors and the reviewers. Any product that may be evaluated in this article, or claim that may be made by its manufacturer, is not guaranteed or endorsed by the publisher.

## Footnotes

1
https://www.arabidopsis.org/

2
https://blast.ncbi.nlm.nih.gov/Blast.cgi

## Supplementary Material

Supplementary Figure 1 Phylogenic relationships of the 29 *Arabidopsis* PLAs and their homologs in *Brassica* species (*B. rapa*, *B. oleracea*, and *B. napus*). The homologs were identified by blasting *Arabidopsis* peptide sequences against *Brassica* peptide sequences using the BLASTP tool (*E*-value ≤1e–10, coverage >60%, identity >60%, and the top 20 hits). The phylogenic tree was constructed with fasttree using PLA peptide sequences aligned by MUSCEL in Mega7. *Arabidopsis* WKRY2 (AT5G56270) was used as the root. Each PLA encoding gene in *B. rapa* was followed with an *Arabidopsis* gene ID in the genome annotation, which was kept in the phylogenic tree to confirm the clustering of the PLAs.

Supplementary Figure 2 Expression changes of *BnPLAs* in resistant and susceptible *Brassica napus* following *Plasmodiophora brassicae* inoculation. **(A)** The expression changes of plants in response to *P. brassicae* pathotype 3A (Zhou et al., 2020b). **(B)** The expression changes of plants in response to *P. brassicae* pathotype 5X (Galindo-González et al., 2020).
