# Supplementary figures and images for: Identification of Arabidopsis Phospholipase A Mutants With Increased Susceptibility to Plasmodiophora brassicae
Source: Front Plant Sci. 2022 Feb 18;13:799142. doi: 10.3389/fpls.2022.799142 (PMC8895301; doi:10.3389/fpls.2022.799142)

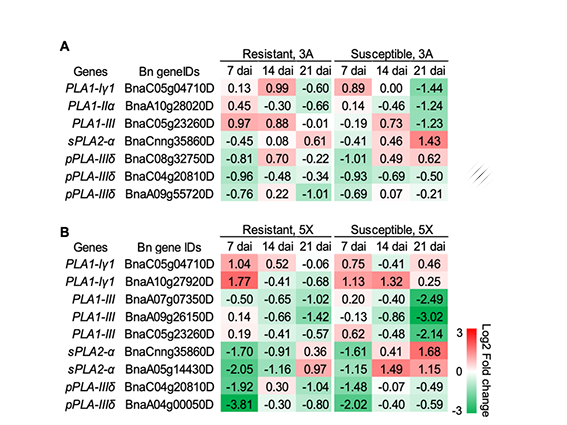

Supplement: Supplementary Figure 1 — Phylogenic relationships of the 29 Arabidopsis PLAs and their homologs in Brassica species (B. rapa, B. oleracea, and B. napus). The homologs were identified by blasting Arabidopsis peptide sequences against Brassica peptide sequences using the BLASTP tool (E-value ≤1e–10, coverage >60%, identity >60%, and the top 20 hits). The phylogenic tree was constructed with fasttree using PLA peptide sequences aligned by MUSCEL in Mega7. Arabidopsis WKRY2 (AT5G56270) was used as the root. Each PLA encoding gene in B. rapa was followed with an Arabidopsis gene ID in the genome annotation, which was kept in the phylogenic tree to confirm the clustering of the PLAs. [file Image_1.TIF]

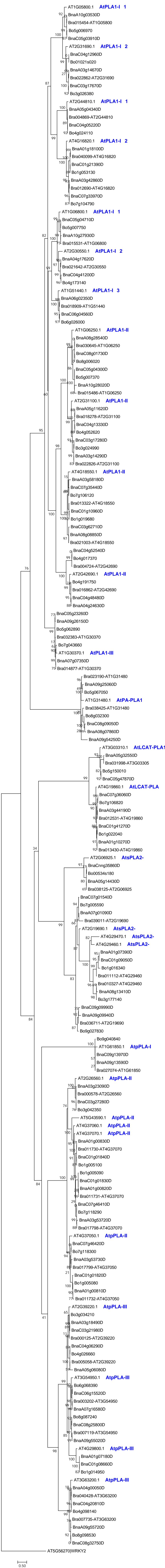

Supplement: Supplementary Figure 2 — Expression changes of BnPLAs in resistant and susceptible Brassica napus following Plasmodiophora brassicae inoculation. (A) The expression changes of plants in response to P. brassicae pathotype 3A (Zhou et al., 2020b). (B) The expression changes of plants in response to P. brassicae pathotype 5X (Galindo-González et al., 2020). [file Data_Sheet_1.PDF]
